# Environmental Intervention in a University Canteen with Focus on Decision Guidance—Effects on Sale and Daily Consumption of Vegetables and Fruit

**DOI:** 10.3390/ijerph181910443

**Published:** 2021-10-04

**Authors:** Melanie Schneider, Carolin Nössler, Petra Maria Lührmann

**Affiliations:** Institute of Health Sciences, University of Education Schwäbisch Gmünd, Oberbettringer Straße 200, 73525 Schwäbisch Gmünd, Germany; carolin.noessler@ph-gmuend.de (C.N.); petra.luehrmann@ph-gmuend.de (P.M.L.)

**Keywords:** vegetables, fruit, environmental intervention, decision guidance, university canteen, sale of vegetables and fruit, vegetables and fruit consumption

## Abstract

The purpose of the study was to evaluate whether an environmental intervention in a university canteen changes the sale and daily consumption of vegetables and fruit among canteen users. The intervention focused on decision guidance, including a positive incentive and nudging. In a pretest−posttest-design, daily sales data of pieces (pcs) of vegetable components and fruit, as well as the sale per main component (pcs/mc), were assessed. Here, 20 opening days were analyzed, each after the intervention (t1) and in the same period of the previous year (t0). Vegetable and fruit consumption were assessed in a controlled pretest−posttest design (3-day-dietary-record, t0 and t1). The intervention group (IG; *n* = 46) visited the canteen ≥ once/week, and the control group (CG; *n* = 49) < once/week. At t1, the sale of absolute vegetable components did not change (t0: 132.3 ± 49.7 pcs, *p* > 0.05), but more per main component were sold at t1 (t0: 0.54 ± 0.09, Δ: 0.09 ± 0.13 pcs/mc, *p* < 0.05). In addition, the sale of fruit (t0: 17.4 ± 11.6, Δ: 8.3 ± 10.8 pcs, *p* < 0.05; t0: 0.07 ± 0.03, Δ: 0.05 ± 0.07 pcs/mc, *p* < 0.001) increased after the intervention. The total consumption of vegetables (IG, t0: 260 ± 170 g/d, CG, t0: 220 ± 156 g/d; *p* > 0.05) and fruit (IG, t0: 191 ± 109 g/d; CG, t0: 186 ± 141 g/d; *p* > 0.05), however, did not change. To effectively change daily consumption, the intervention needs to be expanded.

## 1. Introduction

Vegetables and fruit are of particular importance in a healthy diet. An adequate consumption of these foods reduces the risk of disease adjusted life years and premature mortality (e.g., caused by coronary heart disease, stroke, diabetes, and colorectal cancer) [1,2]. But in large groups of the German population [3], such as working people [4] or students [5,6,7,8], vegetable and fruit consumption is often below the recommended five servings per day. Notably, the deficiency in vegetable consumption is high. Therefore, from a health promotion point of view, the consumption of vegetables and fruits should be increased.

Changes in the food environment are appropriate to initiate improvements in dietary behavior [9]. To promote a higher consumption of vegetables and fruit in terms of preventive measures, canteens in the workplace are a suitable setting. In Western societies, university students [10,11] and working people [11,12] often eat lunch away from home. Lunch accounts for approximately 25% of the daily energy intake [13,14]. Furthermore, vegetables are traditionally often eaten for lunch. Therefore, interventions in canteens to increase vegetable and fruit consumption at lunch are promising.

Interventions in order to increase the vegetable and fruit consumption in canteens are often based on the principles of decision guidance. In terms of intervention depth and effectiveness, Jürkenbeck et al. rank decision guidance between decision support (low intervention depth) and decision restriction (highest intervention depth) [15]. Two important elements of decision guidance are nudging and positive incentives (concerning pricing). Nudging avoids the use of prohibitions and economic incentives. A nudge must be relatively easy to circumvent by the recipient [16]. Nudges in canteens can be classified into different types, e.g., proximity (desired behavior can be achieved with less effort) and presentation [17,18]. Most intervention studies in canteens measure the choice/sale [19,20,21,22,23,24,25,26] or the on-site consumption [27,28,29,30,31,32] of vegetables and fruit. To a lesser extent, the influence on the total daily vegetable and fruit consumption of canteen users is examined [33,34,35,36,37]. Even less frequently examined are the coincident effects on choice/sale/on-site consumption and total daily consumption of vegetables and fruit [34,35,37]. However, this enables a better assessment of the possible transfer and compensation effects.

Therefore, the present study aimed to analyze whether a mainly decision guiding environmental intervention in a university canteen impacts the sale and daily consumption of vegetables and fruit. The present intervention study applied different nudging strategies, a positive incentive, and some elements of decision support. It was the hypothesis that the sale of vegetables and fruit would increase. In addition, it was assumed that there were no compensation effects. Consequently, an increase in vegetables and fruit consumption was hypothesized.

## 2. Materials and Methods

### 2.1. Study Design

**Figure 1:**
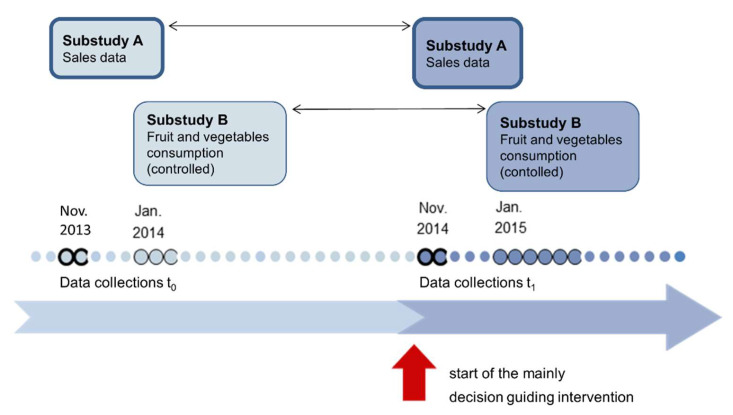
Study design.

Figure 1 displays the study design to evaluate the effects of the mainly decision guiding intervention on the sale of vegetables and fruit in the canteen (Substudy A), and the consumption of vegetables and fruit of the university members (Substudy B). The intervention was implemented in the university canteen at the University of Education Schwäbisch Gmünd, beginning in November 2014. Substudy A was conducted based on a pretest−posttest-design. For the measurement of possible changes in the vegetable and fruit consumption of the canteen users (Substudy B), a dietary assessment was performed using a controlled pretest−posttest-design with a paired sample.

### 2.2. Frame and Intervention

The daily lunch offered in the university canteen comprised four main components. Additional side dishes could be selected, e.g., vegetable components (raw salad, salad buffet, and cooked vegetables) and fruit pieces (piece of fresh fruit and fresh fruit cup). Vegetable and fruit side dishes were already available daily at t0.

The intervention consisted of different measures, according to Jürkenbeck et al. [15], with a focus on decision guidance. It was carried out as part of the implementation of the German Nutrition Society’s Guidelines on Quality Standards for Canteens in the Workplace (DGE-GQS) [38]. DGE-GQS provides information on how to compose a healthy menu, which has to be implemented in at least one menu. Therefore, one menu was reformulated (see Table 1). The university canteen was awarded the highest certification (PREMIUM-Certification) of the German Nutrition Society for implementing these guidelines. Table 1 gives an overview of the measures implemented. The university canteen proposed a health-promoting menu, which included a reformulated main component in addition to the usual offering, as well as a vegetable component and a dessert (fresh fruit at least twice a week) as a suggested combination.

### 2.3. Assessment Methods of Substudy A

Sales data (electronic cash: Toshiba IBM SurePOS 500) regarding vegetable components (raw salad, salad buffet, and cooked vegetable) and fruit (piece of fresh fruit and fresh fruit cup), as well as vegetable components and fruit sold per main component, were assessed. The analysis covered 20 opening days, beginning one week after the intervention started (t1: 17 November–12 December 2014) and in the same period of the previous year (t0: 18 November–13 December 2013).

### 2.4. Assessment Methods of Substudy B

The assessment took place before (t0: 20 January–28 February 2014) and at least ten weeks after (t1: 15 January–17 April 2015) the intervention started. All university members (students and employees ~2800) were invited to participate in the study by e-mail, via the learning management system, by flyer, during lectures, or by personal contact. A minimum of 34 participants was planned according to the tables of Bortz and Döring [40] for repeated measures analysis of variance (assumptions: statistical power of 80%, α = 0.05, δ = 0.5, correlation of $\bar{\rho }$ = 0.50 between measurement series). At t0 187 university members accepted the invitation to participate and 122 at t1. Complete data sets in a paired sample were available for 95 participants. University members who ate a hot lunch in the canteen at least once/week were assigned to the intervention group. Participants with a lower frequency (than one hot lunch a week in the canteen) were allocated to the control group.

The dietary assessment was conducted by a validated food record. Participants recorded their food consumption over three days by estimating the amounts via typical household measures (e.g., spoon) and documented it in a closed form [41].

Additional items were collected by questionnaire (online/paper−pencil). These were gender, age, weight, height, personal presence at university, the use of the canteen for lunch, and the canteen’s health-promoting lunch option. Body mass index (BMI) was calculated via dividing weight (kg) by height in meters-squared.

### 2.5. Statistical Analysis

Statistical analyses were performed using SPSS Statistics version 27 (IBM Corp., Chicago, IL, USA). Comparisons between groups (t0 and t1, Substudy A; intervention group and control group, Substudy B) were conducted using Mann–Whitney U-tests for continuous variables and Pearson’s Chi^2^-tests for categorical variables.

To detect possible changes in the vegetable and fruit consumption over time, an analysis of variance (ANOVA) with repeated measures was performed and the intervention status (intervention group vs. control group) was applied as a covariable. Differences were considered as statistically significant when *p*-values were < 0.05.

### 2.6. Ethical Consideration

The study was performed in accordance with the guidelines of the Declaration of Helsinki. It was approved by the Ethics Committee of the University of Education Schwäbisch Gmünd. Only data of participants who had given their written informed consent were analyzed.

## 3. Results

### 3.1. Substudy A

**Figure 2:**
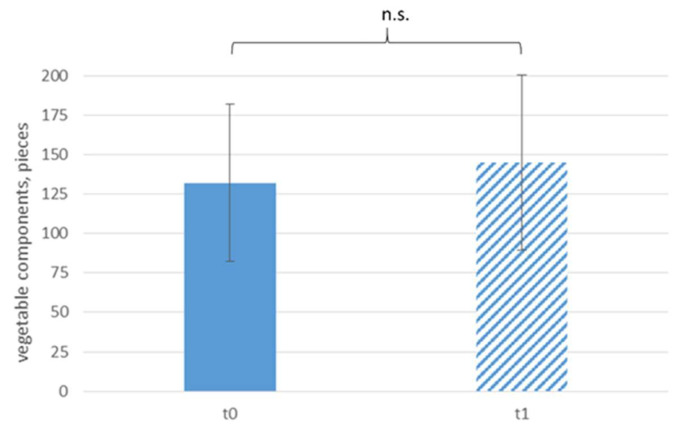
Daily sale of vegetable components according to time; n.s. *p* > 0.05.

**Figure 3:**
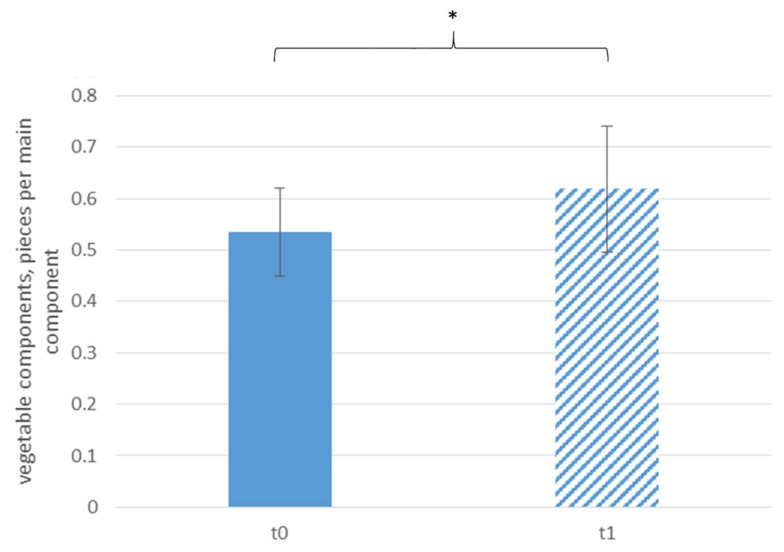
Daily sale of vegetable components per main component according to time; * *p* < 0.05.

Figure 2 displays the daily sale of vegetable components according to time. The sale of vegetable components did not increase significantly after the intervention. However, per main component (mc), significantly more pieces (pcs) of vegetable components were sold at t1, which was an increase of almost 20% (t0: 0.54 ± 0.09 pcs/mc, t1: 0.62 ± 0.12 pcs/mc, see Figure 3).

**Figure 4:**
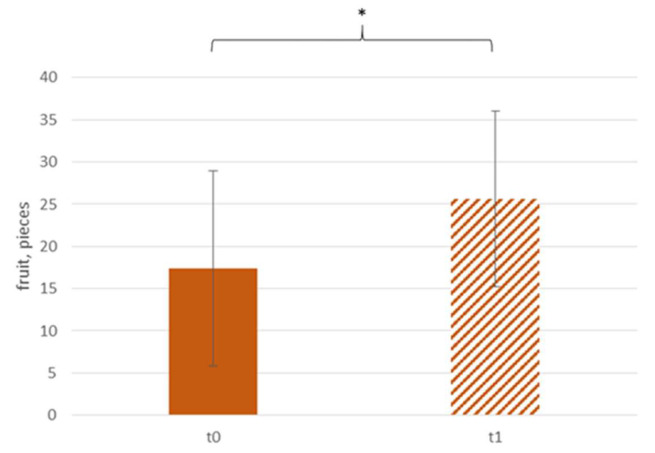
Daily sale of fruit according to time; * *p* < 0.05.

**Figure 5:**
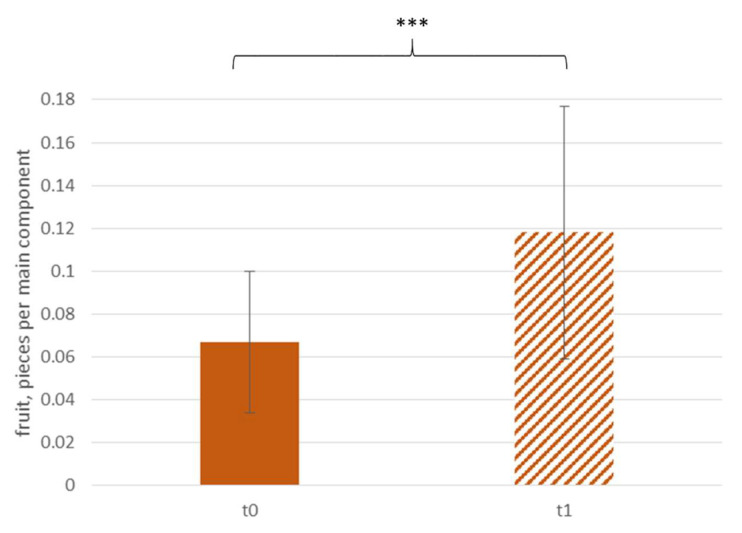
Daily sale of fruit per main component according to time; *** *p* < 0.001.

Figure 4 shows that the sale of fruit increased by approximately 50% (t0: 17.4 ± 11.6 pcs, t1: 25.6 ± 10.4 pcs) after the intervention. Per main component, 0.05 ± 0.07 more pieces of fruit were sold, compared with t0 (t0: 0.07 ± 0.03 pcs/mc, t1: 0.12 ± 0.06 pcs/mc, see Figure 5).

### 3.2. Substudy B

In total, 95 university members participated in Substudy B. The characteristics of the participants according to group are shown in Table 2.

**Figure 6:**
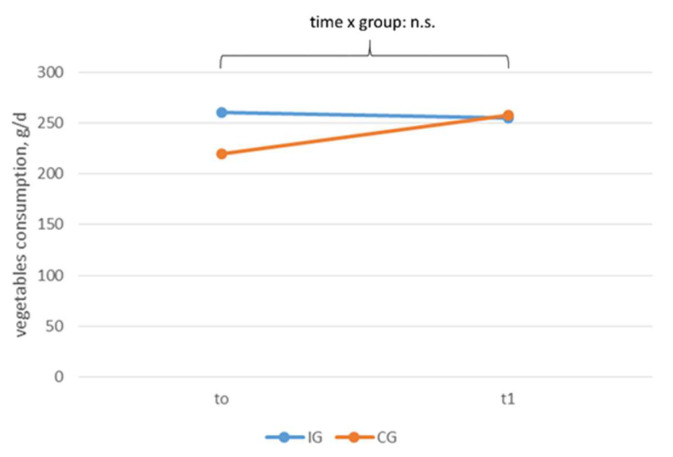
Vegetable consumption according to time and group, n.s. *p* > 0.05.

**Figure 7:**
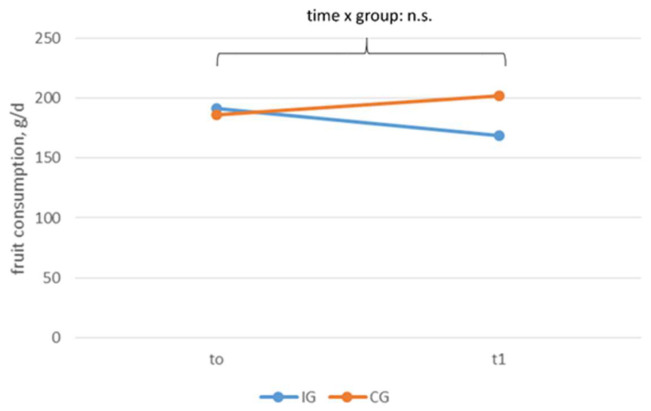
Fruit consumption according to time and group, n.s. *p* > 0.05.

Figure 6 and Figure 7 show the vegetable and fruit consumption according to time and group. At t0, the participants ate about 250 g of vegetables (intervention group: 260 ± 170 g/d, control group: 220 ± 156 g/d) and nearly 200 g of fruit (intervention group: 191 ± 109 g/d, control group: 186 ± 141 g/d) per day. The vegetable and fruit consumption did not change significantly over time. There were no effects regarding time, group, or time x group.

## 4. Discussion

### 4.1. Measures Implemented and Comparative Studies

The hypotheses were partially confirmed. The intervention resulted in higher sales of vegetable components per main component, as well as more fruit in absolute terms and per main component. The hypothesis regarding the consumption behavior could not be confirmed. Unfortunately, the effects on vegetable and fruit sales were relatively small. These effects were hardly measurable or compensated in regard to daily vegetable and fruit consumption.

The present study confirms other studies on the selection behavior/sale or on-site consumption of vegetables and fruit. Apparently, the selection/sale [21,22,26] or on-site consumption [27,29,31] of vegetables and fruit—and especially fruit [19,20,21,22,23,24,25,26,28,30]—can be increased. Often, however, the increases are of little relevance to the actual consumption. Especially in larger randomized controlled trials, the increases tend to be small [24,25,27,28]. In addition, other components sold, e.g., main components, confectionery, and snacks, have an even higher volume [19,20,21,22,26], which is also confirmed in this study. Even after the intervention started, only 0.6 vegetable components and 0.1 pieces of fruit were sold per main component. Future measures to increase sales of vegetables and fruit should therefore be intensified and should include a wider range of products (e.g., through increased variety, nudging, and positive incentives for all vegetable components and fruit).

Particularly high price reductions (>30%) [20,21] or the free offer of vegetables and fruit [35] lead to significantly higher sales or consumption of vegetables and/or fruit. A price reduction on a specific bundle of products including vegetables and/or fruit was less successful in this study, as well as in Velema et al. [24]. Simply designed price reductions and reductions of at least 20% [42] should be applied more frequently. It is not without reason that price incentives are ranked above nudging and decision support in the hierarchy of measures [15]. In the case of nudging and decision support, the combination and intensity of the measures are relevant and lead to differing results accordingly. In particular with regard to vegetables, increases in selection/sales [19,24,25], as well as on-site consumption [28,30,32,37], are not always identified.

Another strategy used in the present study, increasing the vegetable content in the main component (reformulation), seems to be very promising, at least in the laboratory. Increasing the proportion of vegetables in a main component is often not noticed by test persons [43,44], yet leads to acceptable ratings of the taste of the main component [43,44] and a significantly higher vegetable consumption [43]. In field studies, vegetable and fruit consumption [27,31] also increased in the canteen. However, the measure’s success strongly depends on the proportion of reformulated meals in the total offer. In the present study, this—one reformulated main component out of a total of four main components—was too small to increase the total daily vegetable consumption. Likewise in this case, the increase in vegetable content should include all main components offered.

Regarding the daily vegetables and fruit consumption, no effects were confirmed. Reasons for this can only be speculated. Whether the differences between the intervention group and control group play a role remains unclear. It seems unlikely that this is a possible reason, as vegetable and fruit consumption increased slightly in the control group while remaining at a similar level in the intervention group. The measures were not effective enough, possibly because they covered too few of the meals offered. In addition, the study included a relatively small number of participants, and the methods could have been slightly more precise.

The outcome parameter of the total daily vegetable and fruit consumption has only been used in a few intervention studies in canteens [33,34,35,36,37]. Effects on daily vegetable consumption [33,34] or vegetable and fruit consumption [35] were only found in studies with a high intervention intensity [35] and high intervention exposure [33,34,35]. More research is needed, particularly concerning the rather inadequate consumption of vegetables (compared with fruit) and the mixed results from studies with preliminary parameters (selection behavior/sale or on-site consumption of vegetables). Randomized, controlled studies, which are more complex and elaborate, would be ideal, yet are currently scarce [35,37] and are designed for the medium- [37] or long-term.

### 4.2. Strength and Limitations

One limitation of the present study is that the analysis of the sales data (Substudy A) is unfortunately only based on a pretest−posttest design. However, this is also the predominantly used design in other studies [19,20,21,22,23,26] on selection behavior/sales. A controlled design would be more promising. In turn, the already relatively long intervention period hinders the recruitment of a number of canteens for such an intervention [45]. One advantage of using sales data over on-site consumption data is that the entire breadth of canteen users is represented (no selection bias). Moreover, sales figures refer to a naturally found field, confirming the feasibility in contrast to artificially constructed experimental procedures in the field [35].

On a positive note, Substudy B provides the controlled study design mentioned above. Although there are some differences between the intervention group and control group, both groups came from the same social environment of a university. Ideally, a long-term RCT with simultaneous measurement of sales or on-site consumption and total daily consumption would have been conducted.

Admittedly, the number of participants in this study was relatively small, and the drop-out rate did not meet the optimal maximum of 20% for nutrition-related intervention studies in general [46]. However, some drop-outs due to graduation could not be avoided and the drop-out rate was lower than in the well conducted RCT by Steenhuis et al. [37], with a shorter follow-up duration.

The intervention group in this study had relatively low exposure to the intervention (2.3 ± 1.2 canteen visits/week), which represented below-average canteen use among students [10], the largest group among participants, and was a counter to strong intervention effectiveness. Finally, the precision of the measured vegetable and fruit consumption could be improved. It is possible that hidden vegetables were not noticed [43,44] and were not sufficiently documented in the 3-day estimated food record. However, at least the chosen method provided consistent data quality.

## 5. Conclusions

Through the intervention, the sales of vegetables (per main component) and fruit (absolute and per main component) increased to a small extent. This seems easier to achieve for fruit than for vegetables. However, for vegetables, there is a greater requirement (greater consumption deficit compared to the recommendation) and less clear study evidence. Nevertheless, the total daily consumption of vegetables and fruit could not be increased.

In the present study, the measures were centered on the marketing of a health-promoting menu. Expanding reformulation, nudging, and pricing incentives to a broader range of products is recommended. The more courageous application of these measures also offers the opportunity to bring about changes in overall daily consumption. It can be assumed that only the exclusive application of the measures to the entire lunch offer will have a relevant impact on daily vegetable and fruit consumption. Moreover, the effect could unfold across social differences in all social groups [47]. This should be verified in further studies.

## Figures and Tables

**Table 1:** Measures implemented in the present study according to Jürkenbeck et al. [15].

Concept According to Jürkenbeck et al. [15]		Measures of the Intervention in the Present Study
**Decision Restriction**	**Limited selection through product bans**	
	**Limited selection through product reformulation and governmental product standards**	One traditional main component was reformulated according to DGE-GQS [38], resulting in a higher content of vegetables (+48 ± 68 g/main component, *p* < 0.01), but not of fruit (*p* > 0.05) [39]. It was the core item of the health-promoting menu.
**Decision Guidance**	**Guided selection through negative incentives**	
	**Guided selection through positive incentives**	If guests selected the health-promoting menu as suggested by the university canteen, they received a discount of 20% off the menu price.
	**Guided selection through nudging**	Fruit was presented in a more attractive fruit bowl (presentation). The health-promoting menu was particularly highlighted in a showcase and also on the daily menu (presentation). The health-promoting menu components were all offered at one counter (proximity; usually the main components and side dishes were served at different counters). One health-promoting menu, consisting of a reformulated main component (see above) and health-promoting side dishes (including vegetables and fruit), was available daily (availability). The canteen staff at the counter pointed out how to compose the health-promoting menu (prompting).
**Decision Support**	**Simplified choice**	The components of the health-promoting menu were labeled with a STUDY&FIT-Logo (labeling). Fruit was always labeled with a STUDY&FIT-Logo (labeling).
	**Informed choice**	Information material was provided about a healthy diet and the health-promoting menu (e.g., poster, leaflets, and online information), including nutritional value.
	**Governmental unregulated choice**	The rest of the food offers, besides the health-promoting menu (three out of four main components and different side dishes), were not regulated

**Table 2:** Characteristics of study participants according to group.

	Intervention Group		Control Group		*p* ^†^
	*n*	% or Mean ± SD	*n*	% or Mean ± SD	
Status:					0.047
university student employee	27 19	58.7% 41.3%	39 10	79.6% 20.4%	
Gender:					0.012
Male female	11 35	23.9% 76.1%	2 47	4.1% 95.9%	
Age, years	46	30.8 ± 12.8	49	26.5 ± 9.4	0.453
BMI, kg/m^2^	46	23.4 ± 3.0	48	21.2 ± 2.6	0.000
Personal presence at the university, times/week ^‡^	46	3.8 ± 0.9	49	3.0 ± 1.3	0.002
Canteen visits for lunch, times/week ^‡^	46	2.3 ± 1.2	49	0.2 ± 0.2	0.000
Use of the health-promoting menu, times/week ^‡^	35	0.7 ± 0.9	38	0.1 ± 0.2	0.000
Use of the health-promoting menu, per canteen visit, % ^‡^	35	26.6 ± 27.9	36	19.3 ± 35.5	0.031

^†^ Mann−Whitney U-tests were used for differences in participant’s characteristics across intervention group and control group and Pearson’s Chi^2^-Test for categorical variables, ^‡^ at t1.

## Data Availability

The data presented in this study are available upon request from the corresponding author. The data are not publicly available due to privacy.
